# Delayed Adjacent Level Spondylodiscitis after Initial Surgery with Instrumented Spinal Fusion: A Report of Three Cases and Review of the Literature

**DOI:** 10.1155/2018/4645906

**Published:** 2018-01-18

**Authors:** Narihito Nagoshi, Masanobu Shioda, Etsuro Yorimitsu, Mitsuru Yagi

**Affiliations:** ^1^Department of Orthopedic Surgery, National Center for Musculoskeletal Disorders, National Hospital Organization, Murayama Medical Center, 2-37-1 Gakuen, Musashimurayama, Tokyo 208-0011, Japan; ^2^Department of Orthopaedic Surgery, Keio University School of Medicine, 35 Shinanomachi, Shinjuku, Tokyo 160-8582, Japan; ^3^Department of Orthopedic Surgery, Shioda Memorial Hospital, 550-1 Kori, Choseigun Nagaramachi, Chiba 297-0203, Japan; ^4^Department of Orthopedic Surgery, Hino Municipal Hospital, 4-3-1 Tamadaira, Hino, Tokyo 191-0062, Japan

## Abstract

To date, 2 cases of adjacent level spondylodiscitis occurring a few months after initial spinal fusion were reported. However, the development of delayed adjacent level spondylodiscitis is very rare. The authors report 3 cases of spondylodiscitis that occurred at the proximal adjacent level of the fused spine more than 1 year after the initial surgery. Antibiotic treatment was initially chosen in all three cases. In two of the cases, progressive neurological deficit occurred at the level of the infection due to compression of neural elements and spinal instability. For these patients, additional spinal fusion was performed. In each of the three cases, the selected treatment resulted in successful bony fusion at the level of the spondylodiscitis. According to the National Nosocomial Infections Surveillance System, deep wound infection is defined as occurring within 1 year after surgery with instrumentation. The spondylodiscitis in the present cases occurred more than 1 year after the initial surgery, suggesting that these cases may be considered as adjacent segment disease rather than surgical site infection.

## 1. Introduction

Postoperative spondylodiscitis occurring at the surgical level is a well-known complication following instrumented spinal fusion. However, spondylodiscitis occurring at levels adjacent to that of the initial operation is rare. To our knowledge, only two cases of adjacent spondylodiscitis following instrumented spinal fusion have been reported [1, 2]. In these cases, the spondylodiscitis at the adjacent level occurred within a few months following the initial surgery, as a result of surgical site infection (SSI).

In the present cases, the patients developed spondylodiscitis at the adjacent level more than one year after the initial spinal surgery, and successful treatment was achieved using conservative and surgical approaches.

## 2. Case Reports

### 2.1. Case 1

**Figure 1:**
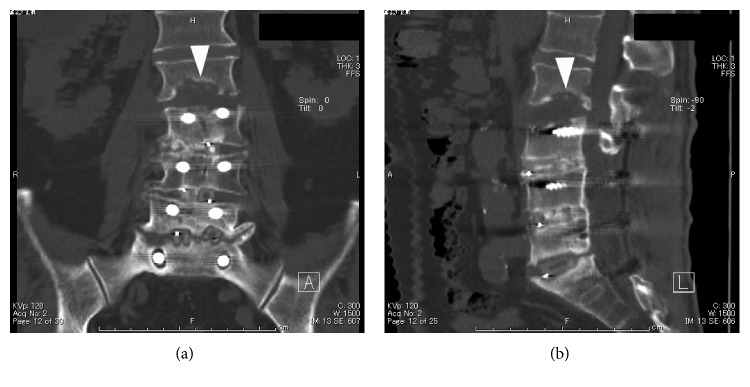
Case 1: coronal (a) and sagittal (b) CT scans showed erosion of endplates at the L2-3 level (arrowhead).

**Figure 2:**
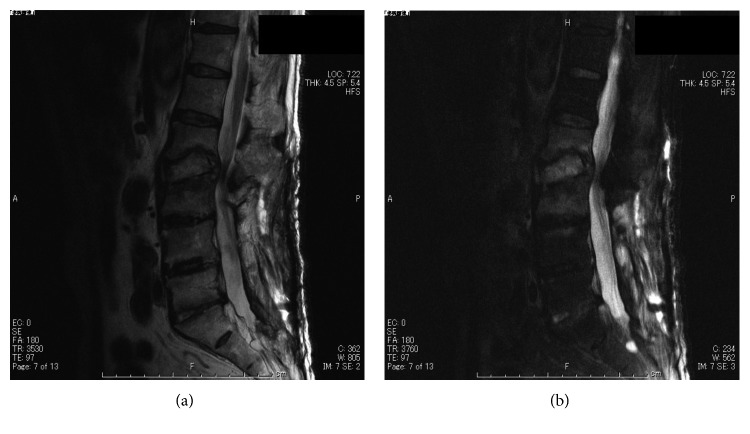
Case 1: plain sagittal T2-weighted (a) and gadolinium-enhanced T1-weighted (b) MRI showed the presence of an abscess at the L2-3 level after removal of the implant.

**Figure 3:**
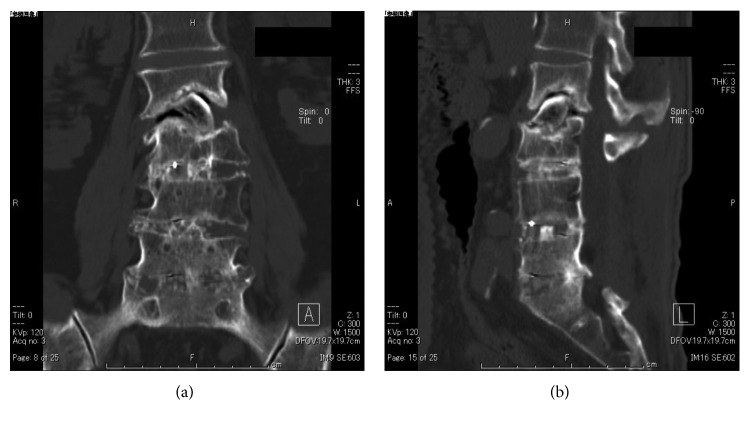
Case 1: coronal (a) and sagittal (b) CT images showed bone union at the L2-3 level 13 months after initial ASF.

A 68-year-old man had undergone posterior lumbar interbody fusion (PLIF) at the L3 to S1 levels for the treatment of lumbar canal stenosis. Bone union was achieved between L3 and L5, but not between L5 and S1. The patient experienced mild lower back pain, which was assumed to originate from pseudoarthrosis at the L5-S1 level. Twenty-one months after the initial surgery, the patient presented with intense lower back pain and was admitted to our hospital. Hematological investigation revealed an increase in both C-reactive protein (CRP) (2.7 *µ*g/ml) and total white blood cell (WBC) count (8.8 × 10^9^/l). Radiographs and computed tomography (CT) scans showed decreased disc height at the L2-3 level, with erosion of endplates (Figure 1), and magnetic resonance imaging (MRI) showed features of spondylodiscitis, with an abscess at the same level. Surgical debridement was performed, and the original implant was removed. Pus was observed at the pedicle screw holes at the L3 level, and methicillin-resistant *Staphylococcus auricularis* (MRSA) was detected in the culture specimen. Intravenous vancomycin (Vancomycin, Mylan, PA) was initiated; however, epidural abscesses remained, and progressive motor weakness of the iliopsoas and quadricep muscles was detected in the left lower limb (Figure 2). Anterior spinal fusion (ASF) with iliac crest autograft at the L2-3 disc space was then performed (Figure 3). Thirteen months after the final surgery, radiographs showed solid bony fusion at the L2-3 level and even at the L5-S1 level. The patient was no longer experiencing lower back pain at the final follow-up.

### 2.2. Case 2

**Figure 4:**
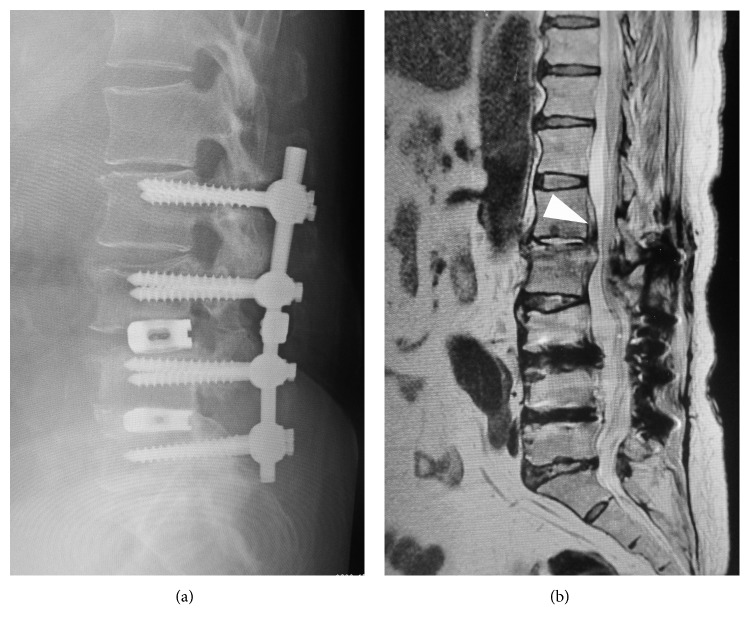
Case 2: plain lateral radiograph (a) and sagittal T2-weighted MRI (b) showed decreased disc height and the presence of an abscess in the disc and extradural space (arrowhead) at the L1-2 level.

**Figure 5:**
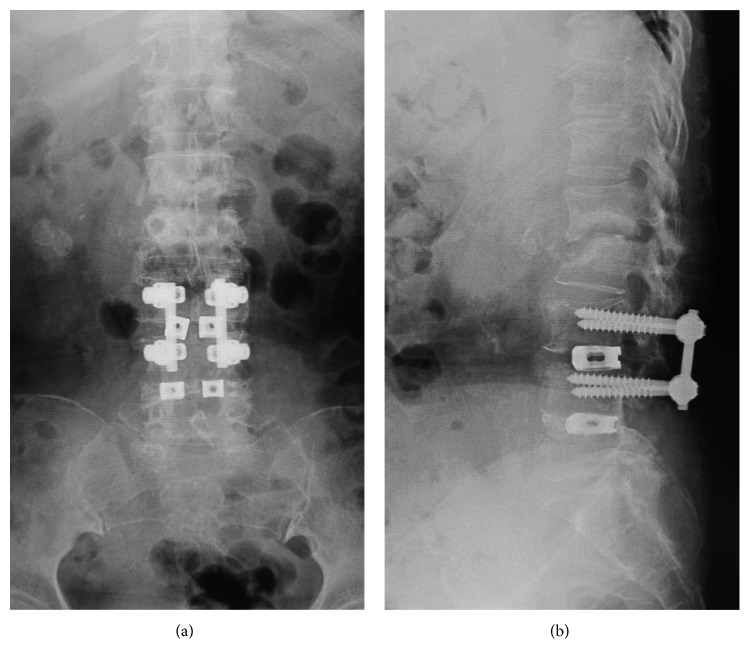
Case 2: plain anteroposterior (a) and lateral (b) radiographs showed progressive vertebral destruction at the L1-2 level after partial removal of the implant.

**Figure 6:**
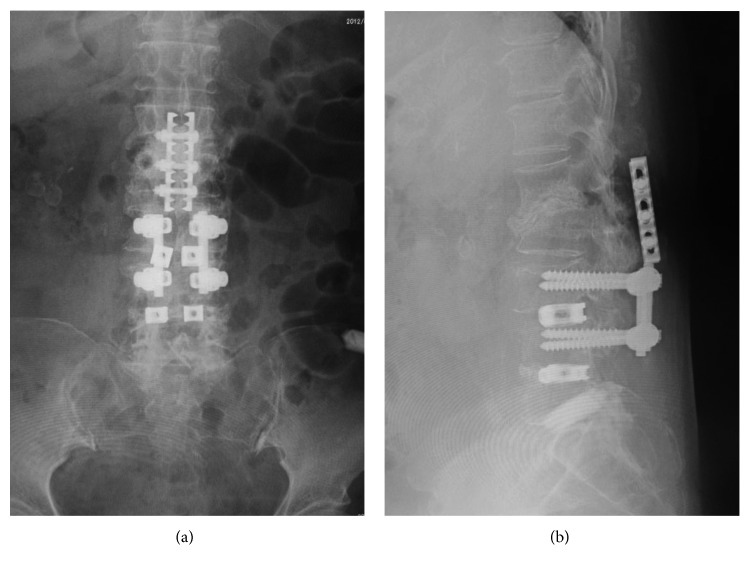
Case 2: plain anteroposterior (a) and lateral (b) radiographs following the final surgery with a spinous process plate and iliac crest bone graft.

A 59-year-old man had undergone posterolateral fusion (PLF) at the L2-3 level and PLIF at the L3–5 levels for the treatment of lumbar canal stenosis. Sixty-eight months later, the patient presented with sudden and intense lower back pain. Hematological investigation revealed an increase in CRP (3.3 *µ*g/ml) and a normal total WBC count (7.40 × 10^9^/l). Radiographs showed decreased disc height at the L1-2 level with erosion of endplates, and MRI showed features of spondylodiscitis with an abscess at the same level and in the extradural space (Figure 4). Despite administration of cefotiam (Pansporin, Takeda, Osaka), erosion of the endplate progressed, and lower back pain continued to increase. Surgical debridement was performed, and the pedicle screws at L2 and L5 were removed. The screws at L3 and L4 were left because pseudoarthrosis was suspected. Pus was observed at the pedicle screw holes at L2, and *Streptococcus intermedius* was detected in the culture specimen. Even after irrigation and debridement, vertebral destruction progressed, resulting in severe instability at the L1-2 level (Figure 5). The patient then underwent ASF with iliac crest autograft at the L1-2 disc space and posterior spine fusion with a spinous process plate. Thirteen months after the final surgery, he was asymptomatic, and the plain radiographs showed bony fusion at the L1-2 level (Figure 6).

### 2.3. Case 3

**Figure 7:**
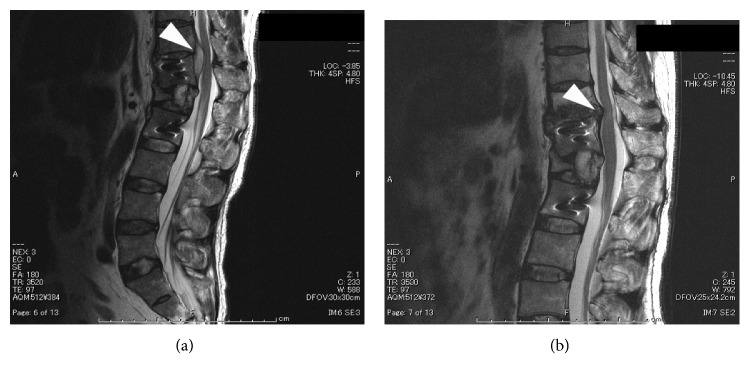
Case 3: sagittal T2-weighted MRI before (a) and after (b) administration of antibiotics. An abscess was observed in (a) and was not detectable in (b).

**Figure 8:**
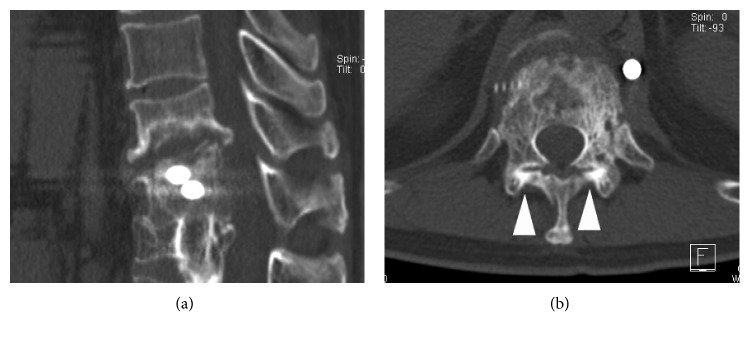
Case 3: sagittal (a) and axial (b) CT scans revealed facet joint union at the T11-12 level (arrowheads).

A 47-year-old man with a history of severe atopic dermatitis had undergone ASF at the T12-L2 levels for the treatment of a burst fracture at the L1 vertebra. Forty-eight months later, the patient experienced intense lower back pain. Hematological evaluation showed increases in both CRP (17.7 *µ*g/ml) and total WBC count (10.5 × 10^9^/l). Radiographs showed decreased disc height at the T11-12 level with erosion of endplates, and MRI showed features of spondylodiscitis with an abscess at the same level and in the extradural space (Figure 7). A culture specimen of the facial skin revealed the presence of methicillin-resistant *Staphylococcus epidermidis* (MRSE), consistent with the patient's history of severe atopic dermatitis. Since the bacteria harvested from the skin were assumed to be the underlying cause of the discitis, conservative treatment was chosen. The patient was treated with intravenous administration of clindamycin (Clindain, Nipro Pharma, Osaka), since MRSE is sensitive to this antibiotic. One month after antibiotic administration, the abscess could not be detected by MRI (Figure 7), and a hematological investigation showed a normal CRP level and total WBC count. Eighteen months after terminating the antibiotics, the patient was in good condition with no symptoms of lower back pain at the final follow-up. The CT scans revealed bone union at the T11-12 level (Figure 8).

## 3. Discussion

A review of the literature revealed two previously reported cases of adjacent spondylodiscitis at the proximal vertebral level [1, 2]. However, in these cases, the spondylodiscitis occurred within 1.5 and 2 months after the primary surgery, respectively. According to the National Nosocomial Infections Surveillance System, deep wound infection is defined as occurring within one year after initial surgery with instrumentation [3]. Thus, the onset of spondylodiscitis in the previously described cases is consistent with the definition of SSI. In contrast, in the three cases in the current study, the adjacent spondylodiscitis occurred more than one year after the initial surgery, thus deviating from the SSI definition. These findings suggest that the current cases may be considered as adjacent segment disease (ASD), rather than SSI.

ASD is known to be caused by degeneration that develops at adjacent mobile segments either proximal or distal to a fused spinal segment. There are several potential risk factors for ASD, and the number of fused segments is one such risk, as the longer lever arm produced by polysegmental fusions causes more stress at the remaining free segments [4]. In one study in which instrumentation was used in patients to promote lumbar fusion for the treatment of degenerative instability, 78% of the patients developing ASD involved the fusion of two or more segments [5]. In all of the present cases, the fusions involved more than two segments, supporting the possibility that the rigidity associated with longer spinal fusion constructs results in greater mechanical stress leading to accelerated degeneration at neighboring segment levels.

The pathogenesis of adult spondylodiscitis involves the occurrence of a septic embolus at the end arteries of the vertebral endplate, that results in a large infarct and subsequent spread of infection [6]. We do not yet have a clear explanation for this delayed adjacent level spondylodiscitis following spinal fusion with instrumentation. However, one possibility is that the concentrated stress occurring at the level adjacent to the spinal fusion results in the development of ASD. Bacteria could tend to remain at the vertebral endplate due to a disturbance in blood flow. Moreover, pedicle screws in the three cases were inserted just below the endplate at the primary surgery, raising the possibility that circulation dynamics in the arterial network of the endplate was disrupted. The disrupted circulation may have led to the formation of a septic embolus, resulting in spondylodiscitis at the adjacent level.

To our knowledge, *Staphylococcus auricularis* spondylodiscitis has not been reported previously. *Staphylococcus auricularis* is coagulase negative and novobiocin sensitive. Most infections due to coagulase-negative staphylococci are nosocomial. Sixty to eighty percent of nosocomial coagulase-negative staphylococci are methicillin resistant. In fact, the *Staphylococcus auricularis* in Case 1 was also found to be methicillin resistant and was sensitive to vancomycin. Therefore, surgical treatment combined with the administration of vancomycin was appropriate and cured the spondylodiscitis. Similar to the second case, two cases of spondylodiscitis caused by *Streptococcus intermedius* were reported previously [7, 8]. *Streptococcus intermedius* is a part of the normal oral flora and is known to be a causative organism for endocarditis [7]. Therefore, the portal of entry of *Streptococcus intermedius* into the bloodstream may be the patient's gums at the time of some trauma.

The patient in Case 3 had severe atopic dermatitis, which had been treated with various antihistamines. *Staphylococcus epidermidis* was identified, and intravenous antibiotics were an effective treatment for the spondylodiscitis. Although we did not attempt to identify the organism from the inflamed spinal area by biopsy or surgery, *Staphylococcus epidermidis* was considered to be the causative agent in view of the clinical response to the antibiotic treatment.

Conservative treatment including antibiotics and orthosis is the first choice for eradicating spondylodiscitis. In fact, the patient in Case 3 was successfully treated by administering antibiotics and using a hard corset. The indication for surgical intervention includes compression of neural elements, spinal instability due to extensive bony destruction, severe kyphosis, or failure of conservative treatment [6]. Muscle weakness due to compression of the cauda equina by an epidural abscess occurred in Case 1, and instability due to bony destruction was observed in Case 2. Therefore, the choice of surgical treatment in these two cases was appropriate.

With regard to surgical approaches, the anterior approach and the combined type using both anterior and posterior approaches were chosen in Cases 1 and 2, respectively. Surgical treatment using the anterior approach consists of full disc removal and careful excision of the affected vertebral bodies, followed by tricortical bone graft, harvested from the iliac crest to fill the cavities. Most authors agree that the Hong Kong procedure of anterior radical debridement with strut graft fusion is superior to other procedures [9–11]. In Case 2, because spinal instability was prominent, posterior fusion with a spinous process plate was added to promote stabilization of the spine. Additional posterior fixation should be considered when patients with spondylodiscitis have severe bone destruction and instability.
